# Quality of Life of Patients Using the Hemodialysis Reliable Outflow (HeRO) Graft in Hemodialysis

**DOI:** 10.7759/cureus.3915

**Published:** 2019-01-19

**Authors:** Muhammad Haisum Maqsood, Kinza Rubab

**Affiliations:** 1 Epidemiology and Public Health, King Edward Medical University / Mayo Hospital, Lahore, PAK; 2 Internal Medicine, King Edward Medical University / Mayo Hospital, Lahore, PAK

**Keywords:** hero graft, hemodialysis, end stage renal disease

## Abstract

End-stage renal disease (ESRD) is one of the most feared consequences of kidney disease. A large number of patients with ESRD require long-term hemodialysis. Vascular access options for hemodialysis include the placement of arteriovenous (AV) fistulas, AV grafts, and tunneled dialysis catheters (TDCs). An alternative to the TDC is the Hemodialysis Reliable Outflow (HeRO; Cryolife Inc., Eden Prairie, MN, USA) Graft. The HeRO Graft has been designed to overcome the development of central venous stenosis or occlusion. The objective is to evaluate the quality of life of patients using the HeRO Graft in end-stage renal disease for hemodialysis. We searched PubMed, Medical Literature Analysis and Retrieval System Online (MEDLINE), Excerpta Medica dataBASE (EMBASE), Cumulative Index to Nursing and Allied Health Literature (CINHAL), Directory of Open Access Journals (DOAJ), Pubpsych, and Google Scholar on October 30, 2018. We included published articles in the English language that used the HeRO Graft for ESRD. The adequacy of dialysis and bacteremia rates proved to be equal to those of conventional AV grafts. It turned out that 2.21 interventions per year were needed to maintain the patency of the HeRO Graft while only 1.17 interventions were needed to maintain the patency of the lower extremity graft. Mortality, ischemia, and infection rates were similar for both groups. The tunneled dialysis catheters have a higher incidence of infection as compared to the HeRO Graft. The initial device and placement costs for the HeRO Graft were higher than those for TDCs but savings from the lower incidence of device complications and longer effective device patency make it cost-effective. Based on the limited evidence, it has been discerned that the HeRO Graft is an optimal option for hemodialysis in patients of ESRD who have exhausted all means of upper extremity access. Though almost similar to the AV grafts in terms of complications and less functional than femoral grafts, it still outclasses them in improving the quality of life of such patients.

## Introduction and background

The condition

End-stage renal disease (ESRD) is one of the most feared consequences of kidney disease. It affects over 500,000 patients in the United States and is increasing in prevalence, with over 100,000 new cases reported each year [1]. ESRD occurs when kidney function has deteriorated to an extent that it is no longer adequate to sustain life, unless renal replacement therapy, dialysis, or transplantation is done [2].

A large number of patients with ESRD require long-term hemodialysis (HD), which is a life-preserving therapy [3]. According to estimates, more than 1.5-million patients receive regular HD treatment worldwide, with the number growing at an annual rate of around 7% [4]. With the increased life expectancy of dialysis patients, the challenges of maintaining dialysis access increase as well [5].

The intervention

Vascular access options for hemodialysis include the placement of arteriovenous (AV) fistulas, AV grafts, and tunneled dialysis catheters (TDCs) [6]. An alternative to the TDC is the Hemodialysis Reliable Outflow (HeRO; Cryolife Inc., Eden Prairie, MN, USA), which was approved by the United States Food and Drug Administration (USFDA) as a graft for use in ESRD in 2008 [6]. Patients with end-stage renal disease who require hemodialysis are at risk for the development of central venous stenosis or occlusion, with an estimated prevalence of 16% to 50% [7-9]. The HeRO Graft has been designed to overcome these limitations, but it typically requires several weeks for tissue incorporation [10].

How the intervention works

The HeRO Graft is entirely subcutaneous. It consists of two primary components: a conventionally expanded polytetrafluoroethylene (ePTFE) graft component and a silicone venous outflow component. The ePTFE graft component is placed in the upper arm and anastomosed to the target artery for arterial inflow, and the venous outflow component is placed similarly to a TDC with its distal end terminating at the cavoatrial junction. Two components are placed entirely subcutaneously. When they are brought together via a titanium connector, it results in the shunting of arterial blood from the donor artery into the central venous system, thereby bypassing the need for creating a formal venous anastomosis for outflow [11-13]. Hence, for patients with no adequate upper extremity peripheral venous outflow, the HeRO Graft can provide an opportunity for a preferred upper extremity subcutaneous AV dialysis graft.

The importance of this review

The absence of reviews that have assessed the quality of life of patients using the HeRO Graft has prompted us to evaluate the available evidence to establish the benefits and harms of the HeRO Graft in relation to different hemodialysis techniques in patients with ESRD.

Objective

To evaluate the quality of life of patients using the HeRO Graft in end-stage renal disease for hemodialysis.

## Review

Methods

Search Methods for the Identification of Studies

A systematic review of the current published literature on the Hemodialysis Reliable Flow (HeRO) Graft for hemodialysis in ESRD, in accordance with Preferred Reporting Items for Systematic Reviews and Meta-analyses (PRISMA), was undertaken. A systemic search for systematic reviews, meta-analyses, multicenter studies, and randomized controlled trials was made using the search terms "HeRO Graft," "Hemodialysis," and "End Stage Renal Disease." We systematically searched the PubMed, Medical Literature Analysis and Retrieval System Online (MEDLINE), Excerpta Medica dataBASE (EMBASE), Cumulative Index to Nursing and Allied Health Literature (CINHAL), Directory of Open Access Journals (DOAJ), Pubpsych, and Google Scholar on October 30, 2018. Nine articles were selected and included in this study. Since most of the existing articles dated back to at least 10 years ago, there was no need to set a time limit. The inclusion and exclusion criteria have been listed in Table 1. The exclusion criteria include unpublished articles, conference articles, commentaries, letters to the editor, and reports in a language other than English. We also excluded those articles that did not mention quality of life issues.

**Table 1:** Inclusion and exclusion criteria HeRO: Hemodialysis Reliable Outflow (Cryolife Inc; Eden Prairie, MN, USA); ESRD: End-stage renal disease

Inclusion Criteria	Exclusion Criteria
Published articles	Unpublished articles
Article text in English	Language other than English
Articles related to the HeRO Graft	Unrelated articles
ESRD patients	Irrelevant patients

Data Extraction and Analysis

No statistical analyses or meta-analyses were conducted. Instead, the existing results and conclusions presented in the reviews were extracted and reported in a systematic format.

Results

**Figure 1:**
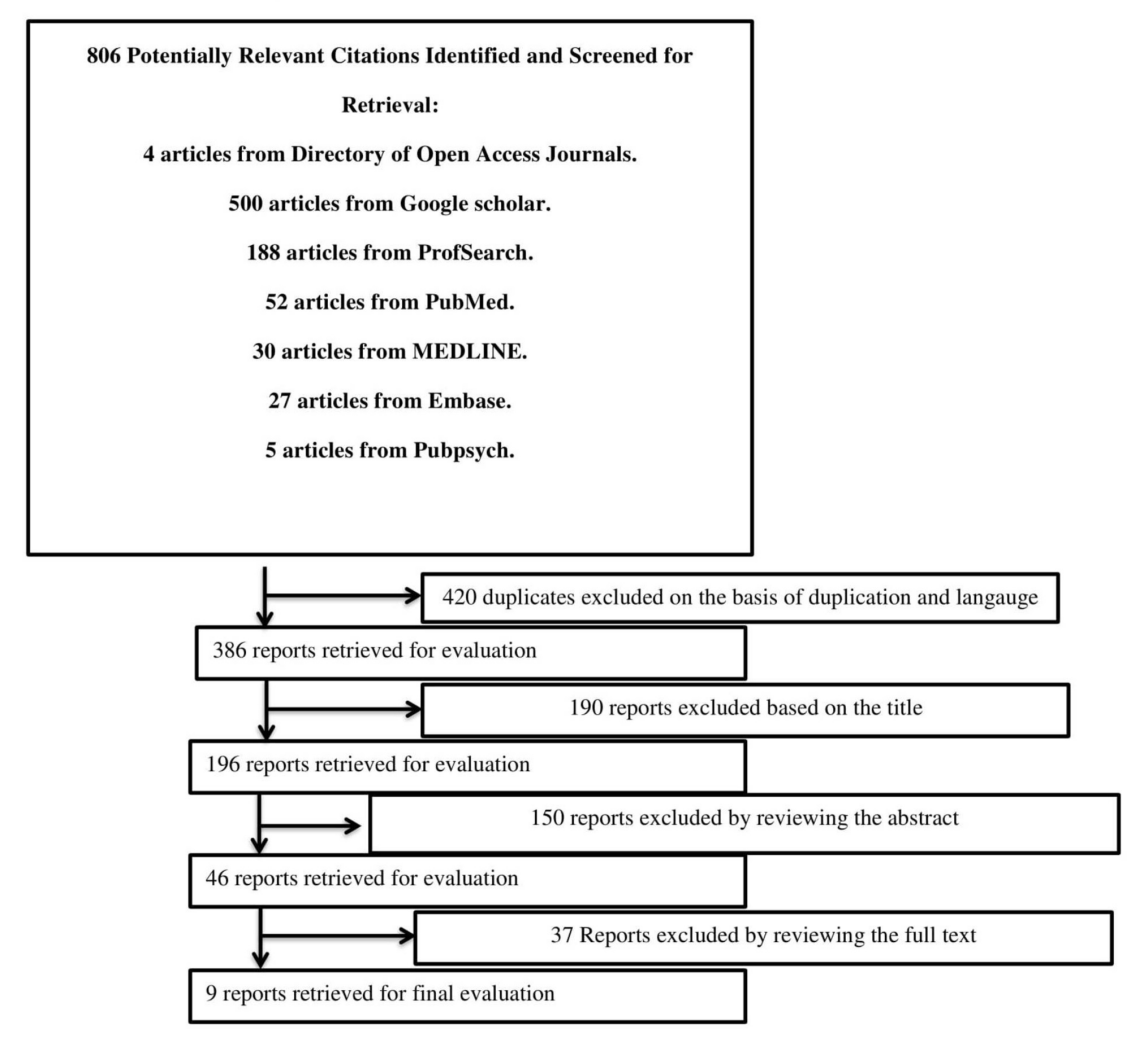
Flow diagram of literature search results

The initial search produced 806 results; 37 qualified for the full-text review. Ultimately, nine were selected, as shown in Figure 1. A quality assessment of the studies was performed per the Grading of Recommendations Assessments, Development, and Evaluation (GRADE) guidelines.

Descriptive analyses/findings

Comparing It to the AV Graft

According to a study included in this review, which involved data collection from 72 subjects (52 HeRO Grafts and 20 AV grafts), primary and secondary patency rates were 34.8% and 67.6% for the HeRO Graft and30.6% and 58.4% in the control group, thus being almost similar. The intervention rates were 2.2/year for the HeRO Graft and 1.6/year for the control (p=0.100). The adequacy of dialysis and bacteremia rates proved to be equal to those of conventional AV grafts [14].

Comparison with the Femoral Graft

According to a study included in this review, it turned out that2.21 interventions per year were needed to maintain patency for the HeRO Graft while only 1.17 interventions were needed to maintain the patency of the lower extremity graft. Mortality, ischemia, and infection rates were similar for both groups. The lower extremity graft had an infection rate of 0.71 per 1000 days and the HeRO had 0.61 per 1000 days. Secondary patency at 12 months for the HeRO Graft was 42% and the thigh graft was 86% [6].

Another study from our review, which had been conducted with 35 femoral AV (fAVG) grafts and 21 HeRO placements showed a primary patency of 40.5%, 18.7%, and 14.9% for fAVG and 29.0%, 29.0%, and 0% for HeRO at six months, 12 months, and two years (p=0.67), respectively. Assisted primary patency was also similar, with 43.8%, 29.4%, and 13.8% for fAVG and 34.8%, 34.8%, and 17.4% for HeRO at six months, 12 months, and two years (p=0.81), respectively. Secondary patency was 62.6%, 50.6%, and 19.3% for fAVG and 68.0%, 53.5%, and 38.3% for HeRO at six months, 12 months, and two years (p=0.69), respectively. The average number of interventions to maintain patency for fAVG was 1.1±1.47 and 1.65±2.52 for HeRO (p=0.35). Infectious rates were 29% in fAVG and 15% in HeRO (p=0.33) [2].

Does It Outclass the Tunneled Catheters?

The results have shown that the tunneled dialysis catheters have a higher incidence of infection as compared to the HeRO Graft and fAVG, suggesting that their use is preferable to catheter dependence [15].

It has been proved in one of the studies included in our reviews that TDCs are the least desirable due to less effective dialysis, an increased risk of thrombosis, higher rates of bacteremia, and greater mortality [16-17].

Infections

Early infection was defined as episodes of bacteremia or HeRO infections requiring resection within 30 days of HeRO implantation. The rate of HeRO Grafts being resected due to infectious complications was 0.41 in directly placed grafts and 0.12 in staged grafts per 1000 implant days [13].

The study of Gage et al. [14] has reported an access-related infection incidence of 0.14/1000 implant days in patients with HeRO, and Katzman et al. [12] have reported a HeRO-related infection incidence of 0.71/1000 implant days.

The Two-staged Technique

In the two-staged technique, the overall rate of infection was 8.6% for primary HeRO implantations and 2.3% for staged implantations (p=.12). The rates of early bacteremia and HeRO resection requiring surgical resection were not significantly different between the groups (p=.19 and p=.065, respectively) [13].

Maintaining the "Real Estate"

Glickman’s study concluded that in patients likely to be on dialysis for more than three to five years, the HeRO device should be considered, as it provides patency rates similar to upper extremity grafts and maintains lower extremity “real estate” [18]. *Cost-effectiveness*

The study in our review (conducted on 100 patients with HeRO Grafts) reported six fewer failed devices, 53 fewer access-related infections, and 67 fewer device thromboses as compared to patients managed with tunneled catheters. The initial device and placement costs for the HeRO Graft were higher than those for TDCs but savings from the lower incidence of device complications and longer effective device patency make it cost-effective [3].

Modified Hero Graft

The HeRO Graft has a drawback: it requires several weeks for tissue incorporation. The ACUSEAL graft (Gore Technologies, Newark, DE, US), a modified HeRO Graft, allows immediate cannulation, thus reducing catheter dependence time and its associated complications. Out of the 10 modified HeRO Grafts placed, postoperative complications included two thromboses and one hematoma. The primary and secondary patency rates were 70% and90%, respectively [11].

Discussion

This systematic review aimed to assess the quality of life of the patients of ESRD using the HeRO Graft for hemodialysis. It included nine studies and has revealed the significant pros and cons of this innovative technique. Among the existing vascular access options, such as conventional AV fistulas, AV grafts, femoral grafts, and tunneled dialysis catheters, the HeRO Graft is a novel addition.

The results of this systematic review signify that the HeRO Graft has patency, rate of infection, intervention, and mortality almost equal to that of a conventional AV graft. But the increased risk of central vein stenosis and occlusion has necessitated the use of an alternative technique such as the HeRO Graft or femoral AV grafts. Though the femoral grafts have higher patency and a low rate of infection and intervention, HeRO Grafts are a better option, as they help preserve the lower extremities for any future access.

Since the HeRO Graft requires time for tissue incorporation in order to get functional, the tunneled dialysis catheters have to be used as an interim. This problem has been addressed with the modified HeRO Graft that requires early cannulation; thus reducing the rate of infection, thrombosis, and the inadequacy of dialysis associated with catheter dependence. The systematic review of ours also suggested that though the initial cost of the HeRO Graft is higher than that of other hemodialysis techniques, the low rate of complications makes it cost-effective.

While valuable information has been gained from this review, a significant issue was the marked heterogeneity among studies about the aspect of the HeRO Graft they tended to focus on. Our review indicates that the results of most of the studies incline toward the HeRO Graft being the optimal option for those patients of ESRD who have exhausted all means of upper extremity access.

The results of our systematic review endorse the already existing renowned studies of Wallace, Gage, Nassar, and Katzman. Our review also revealed a significant paucity of evidence regarding the impacts of the HeRO Graft on quality of life. The existing studies have not systematically evaluated the HeRO Graft, an emerging mode of hemodialysis. Another drawback of the available literature is that it fails to systematically evaluate the complications associated with the HeRO implants. The limitations of the included studies did not enable us to derive conclusive evidence about the adverse outcomes if any.

A limitation of our review is the small number of studies for the systematic analysis and the possibility that relevant studies may have not been included. Some clinically significant parameters have also not been addressed, which is another drawback. The low methodological quality meant that robust conclusions could not be derived. The included studies did not provide sufficient information to assess if they were free of selective reporting. Moreover, we could only find a few parameters to assess quality of life such as infectious complications and vascular access. Despite these limitations, our systematic review holds an important place, as it has been one of the few studies to address the quality of life of patients using the HeRO Graft.

Currently, there is insufficient evidence to establish facts. Suitable clinical research needs to be done to establish the superiority of one hemodialysis technique over the existing ones and to know whether the HeRO Graft is an effective and optimal mode of hemodialysis or not.

## Conclusions

Many of the studies evaluating the HeRO Graft as a mode of dialysis are small, outdated, of poor quality, and have insufficient evidence to establish the role of this mode of hemodialysis. However, it can be concluded that the HeRO Graft excels over the tunneled dialysis catheters but is almost equal to the AV grafts in terms of complications and reinterventions. Though the HeRO Graft is less effective than femoral grafts, it is still an optimal option, as it preserves access to the lower extremities.
